# Disruptions of the olfactory and default mode networks in Alzheimer's disease

**DOI:** 10.1002/brb3.1296

**Published:** 2019-06-04

**Authors:** Jiaming Lu, Qing X. Yang, Han Zhang, Paul J. Eslinger, Xin Zhang, Sichu Wu, Bing Zhang, Bin Zhu, Prasanna R. Karunanayaka

**Affiliations:** ^1^ Department of Radiology The Pennsylvania State University College of Medicine Hershey Pennsylvania; ^2^ Drum Tower Hospital Medical School of Nanjing University Nanjing China; ^3^ Department of Neurosurgery The Pennsylvania State University College of Medicine Hershey Pennsylvania; ^4^ Department of Radiology and Biomedical Research Imaging Center (BRIC) University of North Carolina at Chapel Hill Chapel Hill NC; ^5^ Department of Neurology The Pennsylvania State University College of Medicine Hershey Pennsylvania

**Keywords:** Alzheimer's disease, default mode network, mild cognitive impairment, olfactory fMRI, olfactory network

## Abstract

**Introduction:**

Olfactory deficits are prevalent in early Alzheimer's disease (AD) and are predictive of progressive memory loss and dementia. However, direct neural evidence to relate AD neurodegeneration to deficits in olfaction and memory is limited.

**Methods:**

We combined the University of Pennsylvania Smell Identification Test (UPSIT) with olfactory functional magnetic resonance imaging (fMRI) to investigate links between neurodegeneration, the olfactory network (ON) and the default mode network (DMN) in AD.

**Results:**

Behaviorally, olfactory and memory scores showed a strong positive correlation in the study cohorts. During olfactory fMRI, the ON showed reduced task‐related activation and the DMN showed reduced task‐related suppression in mild cognitive impairment (MCI) and AD subjects compared to age‐matched cognitively normal subjects.

**Conclusions:**

The results provide in vivo evidence for selective vulnerability of ON and DMN in AD and significantly improves the viable clinical applications of olfactory testing. A network‐based approach, focusing on network integrity rather than focal pathology, seems beneficial to olfactory prediction of dementia in AD.

## 1 INTRODUCTION

Impairment in olfaction and memory are among the first clinical symptoms of Alzheimer's disease (AD) (Doty, Reyes, & Gregor, 1987; Waldton, 1974). This is likely due to the close anatomical and functional associations between olfaction and memory systems (Karunanayaka et al., 2017). Impaired odor identification in particular has been found to be predictive of progressive memory loss and dementia characteristic of AD, however, the relationships between olfactory deficits, dementia, and neurodegeneration remain unclear (Doty et al., 1987; Moberg et al., 1987; Murphy, 2019; Rezek, 1987; Roberts et al., 2016). In AD, the primary olfactory cortex (POC), hippocampus, and posteromedial cortical regions are severely affected but no mechanisms have linked pathology across these regions to olfactory deficits in any systematic way. This information is critical to clarify, particularly whether olfactory deficits are reflecting damage mainly to primary and secondary olfactory brain regions, or whether they are linked to spreading secondary effects of damage in memory and higher‐order cortical areas affected by AD (Reichert et al., 2018).

From a neuroanatomical point of view, downstream relays from the primary olfactory cortex are associated with higher‐order neocortical regions such as the orbitofrontal cortex (OFC), parietal and insula cortices and the limbic system (Gottfried, 2010; Price, 1990; Zatorre, Jones‐Gotman, Evans, & Meyer, 1992). Additionally, olfaction is considered an ambiguous human sense; that is, the olfactory network (ON) relies on bidirectional information transfer between the extended olfactory network and other brain networks in order to achieve optimal odor perception (Gottfried, 2010). Thus, deficits in odor detection, discrimination, and identification in AD patients may reflect disruptions to larger networks beyond the ON (Doty, 1991; Serby, Larson, & Kalkstein, 1991).

Memory impairment in AD has been attributed to atrophy measured using magnetic resonance imaging (MRI), in the medial temporal lobe (MTL), but recent data suggest that memory function is also dependent on a set of neocortical regions known as the default mode network (DMN) (Buckner, Andrews‐Hanna, & Schacter, 2008; Buckner et al., 2009; Gottfried, 2010; Raichle et al., 2001). The DMN is a cognitively‐related brain network that is suppressed (deactivated) during successful memory formation and goal directed behavior (Greicius, Krasnow, Reiss, & Menon, 2003; Greicius & Menon, 2004; Smith et al., 2009). Importantly, the DMN is impaired in AD (Zhang et al., 2010). In Karunanayaka et al. (2017), they showed a significant suppression (deactivation) in the DMN during an olfactory fMRI activation task, suggesting that odor processing may be drawing cognitive, attentional, and memory resources (Karunanayaka et al., 2017). Therefore, we predicted that the functional connectivity (FC) between the ON and DMN can provide (a) a neural basis to relate olfactory and memory deficits in AD and (b) a sensitive predictor of olfactory deficits in early AD. Understating the relationship between neurodegeneration, memory and olfactory function is a critical first step toward developing olfactory assessment as a measure of progression to AD dementia.

There is ample evidence to suggest that AD neurodegeneration (atrophy) spreads and causes disruption across neural networks (Ahmed et al., 2016; Bede, 2017; Filippi et al., 2017; Guo et al., 2016). Therefore, this study focused on testing a human model of AD neurodegeneration‐to‐olfactory impairment to evaluate a mechanistic account of how AD progression can concurrently disrupt the integrity of the ON‐DMN network. Network atrophy, likely reflecting the cumulative loss and shrinkage of the neuropil, was measured using volumetric magnetic resonance imaging (MRI) (Jack et al., 2010, 2013, 2018; Zarow et al., 2005). The University of Pennsylvania Smell Identification Test (UPSIT) was used to assess the olfactory function of our study cohort, a consistently used test in studies of olfactory dysfunction in AD (Devanand et al., 2010; Nordin & Murphy, 1996; Vasavada et al., 2017). We hypothesized that progressive neurophysiologic disruption to ON and DMN integrity gives rise to early olfactory deficits, which will provide greater prediction of clinical conversion to dementia. We expected ON‐DMN integrity to be highly dynamic in subjects with mild cognitive impairment (MCI), a transitional stage between normal cognition and dementia (Farias, Mungas, Reed, Harvey, & DeCarli, 2009; Petersen, 2006; Visser, Kester, Jolles, & Verhey, 2006). Specifically, we predicted that the effective connectivity (directional influences) between the ON and DMN would be correlated with behavioral olfactory scores in MCI.

## 2 METHODS

### 2.1 Subjects

Thirty‐one age‐matched cognitively normal subjects [CN; mean age = 69.5 years, 15 females, with a Clinical Dementia Rating (CDR) of 0], 19 MCI subjects (mean age = 72.8 years, 10 females, with a CDR of 0.5), and 12 AD subjects (mean age = 73.7 years, 8 females, with a CDR of 1) participated in the study. CDR uses a 5‐point scale to characterize six domains of cognitive and functional performance and widely used to characterize AD‐related related dementias (Morris, 1997). Subjects were tested with the Mini‐Mental State Examination (MMSE), a 30‐point measure that is used to assess memory and cognitive function in AD (Pangman, Sloan, & Guse, 2000). The Dementia Rating Scale (DRS‐2) was used to assesses the overall level of cognitive functioning (Pedraza et al., 2010). We also used the California Verbal Learning Test (CVLT‐II) to measure episodic verbal learning and memory function, which has been shown to be sensitive to AD pathology (Elwood, 1995; Fox, Olin, Erblich, Ippen, & Schneider, 1998). We recruited primarily from neurology specialty clinics at the Hershey Medical Center with additional outreach programs of the Greater Pennsylvania Alzheimer's Association and Country Meadow Retirement facilities in Hershey, PA, USA. Dr. Eslinger, a neuropsychologist (and a co‐author) reviewed the medical records of every AD and MCI subject. The study was approved by the Institutional Review Board of the Pennsylvania State University College of Medicine. All subjects provided informed consent and were screened to rule out any neurologic or psychiatric conditions other than MCI/AD.

### 2.2 Olfactory testing

Study participants were administered the University of Pennsylvania Smell Identification Test (UPSIT) to assess their olfactory function (Doty, Shaman, & Dann, 1984). In this study, an UPSIT score above 20 was considered hyposmic and a score above 30 was considered normosmic. Subjects with an UPSIT score of <20 retain reduced olfactory perception, but are generally considered anomic. Table 1 provides demographic and cognitive/behavioral test score information for the study cohorts.

**Table 1:** Demographic and behavioral data of the study cohort

	CN (*n* = 31)	MCI (*n* = 19)	AD (*n* = 12)	*F*	*p*	Post hoc tests		
						CN vs MCI	CN vs AD	MCI vs AD
Age	70.40 ± 10.00	72.80 ± 9.40	73.70 ± 12.50	0.60	0.555	NS	NS	NS
Gender (M/F)	16/15	9/10	4/8	0.66#	0.721	NS	NS	NS
GDS	2.94 ± 2.79	6.61 ± 3.75	11.67 ± 6.53	20.98*	*p* < 0.001	*p* = 0.003	*p* < 0.001	*p* < 0.001
CVLT‐II	63.97 ± 13.63	47.32 ± 13.35	21.17 ± 13.22	19.90*	*p* < 0.001	*p* < 0.001	*p* < 0.001	*p* < 0.001
MMSE	28.29 ± 1.64	26.63 ± 1.67	19.25 ± 5.66	18.02*	*p* < 0.001	NS	*p* < 0.001	*p* < 0.001
DRS‐2	141.16 ± 2.38	134.21 ± 7.79	106.75 ± 28.15	30.05*	*p* < 0.001	NS	*p* < 0.001	*p* < 0.001
DRS‐AMSS	12.97 ± 1.60	9.63 ± 3.39	4.08 ± 2.68	30.47*	*p* < 0.001	*p* < 0.001	*p* < 0.001	*p* < 0.001
DRS‐AEMSS	12.26 ± 1.75	8.37 ± 4.34	3.5 ± 2.97	18.68*	*p* < 0.001	*p* < 0.001	*p* < 0.001	*p* < 0.001
DRS‐Memory	24.26 ± 0.77	21.21 ± 2.04	11.67 ± 6.23	35.84*	*p* < 0.001	*p* < 0.001	*p* < 0.001	*p* < 0.001
UPSIT	33.42 ± 4.19	25.58 ± 7.69	11.58 ± 5.42	30.45*	*p* < 0.001	*p* < 0.001	*p* < 0.001	*p* < 0.001

GDS, Global Deterioration Scale; CVLT‐II, The California Verbal Learning Test – second edition; MMSE, The Mini–Mental State Examination; DRS‐2, Dementia Rating Scale 2; DRS‐AMSS, Dementia Rating Scale 2, Age‐Corrected MOANS Scaled core; DRS‐AEMSS, Dementia Rating Scale 2, Age‐ and Education Corrected MOANS Scaled Score; DRS‐Memory, memory portion of the Dementia Rating Scale 2; UPSIT, University of Pennsylvania Smell Identification Test. *, one‐way ANOVA analysis; *p*,* p*‐value; ^#^, chi‐square test; NS, not significant.

### 2.3 Olfactory fMRI task

**Figure 1:**
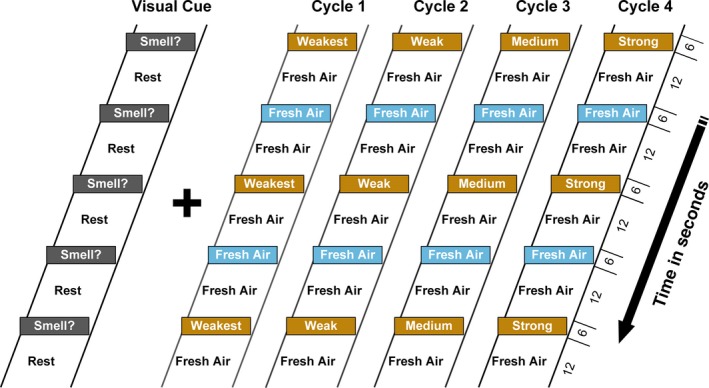
The olfactory fMRI paradigm involving odor–visual association. During odor+visual conditions, the lavender odorant was mixed with moisturized room temperature air and delivered to both nostrils once every 36 s paired with the visual cue, “Smell?”. During visual‐only conditions the same visual cue was presented by itself. The visual cue “Rest” appeared during rest conditions. Four intensities of the lavender odorant were presented. Each odor intensity was presented three times before the next intensity was presented in an ascending order from the weakest to the strongest. To avoid tactile or thermal stimulation, a constant airflow rate of 8 L/min was maintained throughout all conditions, including the “Rest” condition. Participants had to perform a button press response to indicate the presence or absence of an odor during both conditions

The event related, olfactory fMRI paradigm is shown in Figure 1. It consisted of odor+visual and visual‐only conditions in which a constant air flow‐rate of 8 L/min was maintained and also synchronized with the presentation of the visual cue “Smell?” (Karunanayaka et al., 2014, 2015). Four different intensities of the lavender odorant, from weakest to strongest, were used in this task. The odorants were presented for 6 s separated by 30 s of odorless air. The task also included a visual component and a motor response (see Figure 1 for details). This paradigm invokes rapid learning behavior in the olfactory system following odor‐visual pairing. For instance, in Karunanayaka et al. (2015) we showed that the paired visual cue is related to the immediate visually‐evoked activity in the POC, secondary olfactory structures, and the hippocampus. Additionally, we showed that this effect generated no visually evoked odor perception and is also intensity dependent. Since we used four odor intensities in this olfactory fMRI task, it is able to overcome olfactory habituation which has been discussed using a different dataset in Karunanayaka et al. (2014).

The olfactory task, including screen instructions, was clearly explained to study participants before fMRI scanning. Specifically, we made sure that each participant understood the task structure and appropriate button responses. Strict instructions were given to breathe normally during the experiment. During scanning, the technician monitored the respiration patterns of each subject and recorded them via a chest belt connected to the olfactometer. This is to confirm subject wakefulness and normal breathing behavior (rate) during fMRI scanning. The task performance (identifying the presence or absence of an odor) of all subjects were monitored and recorded during fMRI scanning. Subjects who performed above 75% accuracy and had a maximum head motion < 3 mm were included in this study. Prior to scanning, the technician also confirmed that the olfactometer (ETT Olfactometer [Hershey]) and odor delivery/ visual cue presentation were properly working according to the fMRI task timing structure. Optical trigger pulses from the MRI scanner were used to synchronize the odor stimulation paradigm and the MRI image acquisition.

Four intensities of Lavender oil (Givaudan Flavors Corporation, East Hanover, NJ, USA) diluted in 1,2‐propanediol (Sigma, St. Louis, MO, USA) were used as stimuli in the fMRI task due to (a) its effectiveness for olfactory fMRI; (b) minimal propensity to stimulate the trigeminal system and (c) being pleasant and familiar to most North American individuals (Allen, 1936). Olfactory stimuli were stored in six 300 ml glass jars with 50 ml of the odorant.

### 2.4 Imaging parameters

MR images of the entire brain were acquired using GE EPI on a Siemens Trio 3.0 T system with an eight channel head coil. Functional MRI utilizing the blood oxygen level dependent (BOLD) signal was used to evaluate brain functional activities during odor+visual and visual‐only conditions. A T_2_^∗^ ‐weighted echo planar imaging sequence was used to acquire functional data with the following parameters: TR/TE/FA = 2,000 ms/30 ms/90^°^, FOV = 220 mm × 220 mm, acquisition matrix = 80 × 80, 30 slices, slice thickness = 4 mm, and the number of repetitions = 234. For volumetric analysis of the POC and hippocampus, T1‐weighted images with 1 mm isotropic resolution were acquired with MPRAGE method: TE = 2.98 ms, TR = 2300 ms, inversion time (IT) = 900 ms, FA = 9°, FOV = 256 mm × 256 mm × 160 mm, acquisition matrix = 256 × 256 × 160, acceleration factor = 2, and TA = 6 min 21 s.

### 2.5 Data analysis

fMRI preprocessing was done using the SPM8 software (http://www.fil.ion.ucl.ac.uk/spm). Group Independent Component Analysis (gICA) of concatenated data of CN, MCI and AD, was used to identify ON and DMN (Karunanayaka et al., 2014, 2017). The gICA method implemented in this paper consisted of: (a) preprocessing steps (i.e., mean centering and Principal Components Analysis [PCA]) at both individual (40 components) and group (50 components) levels, and (b) ICA decomposition (using the FastICA algorithm), followed by hierarchical agglomerative clustering. Details of this method, particularly in relation to this fMRI task have been explained in Karunanayaka et al. (2014, 2015, 2017). The hemodynamic response function (HRF) and the single‐trial β estimates of each network were evaluated using methods described in Karunanayaka et al. (2017) and Eichele et al. (2008). Demographic and clinical factors were compared using one‐way analysis of variance (ANOVA). The sex ratio between groups was compared with a chi square test. Outlier identification was performed based on values > *M* ± 2.5 *SD*.

### 2.6 Region of interest (ROI) analysis

Primary olfactory cortex and hippocampus were manually segmented as described previously in Vasavada et al. (2015). Briefly, bilateral manual segmentation of the POC and hippocampus was done on T1‐weighted images from each subject using the FMRIB Software Library View (FSLview, Analysis Group, FMRIB, Oxford, UK). The POC included the anterior olfactory nucleus, olfactory tubercle, piriform cortex, anterior portion of the periamygdaloid cortex, amygdala, and anterior perforated substance (Wang et al., 2010). The hippocampus included the hippocampal formation (HF), dentate gyrus, subiculum, parasubiculum, and presubiculum. The segmented POC and hippocampus was performed by trained investigators and reviewed and corrected by a neuro‐radiologist in our lab. Additionally, those who performed the segmentation were blind to group assignment of each subject. The combination of olfactory fMRI, volumetric measurements, and UPSIT scores was intended to develop a method to predict disease onset or used as a tool to monitor disease risk and progression.

### 2.7 Effective connectivity (EC) analysis

In general, MCI subjects show larger variability in behavioral test scores and olfactory fMRI activation profiles (Vasavada et al., 2015). Specifically, MCI subjects' UPSIT scores overlapped with AD and CN subjects, which showed a range of low and high abilities, respectively. Therefore, we expected ON‐DMN connectivity to be highly dynamic in MCI subjects. We investigated this connectivity using the extended unified structural equation modeling (euSEM) (Gates, Molenaar, Hillary, & Slobounov, 2011; Karunanayaka et al., 2014). This EC method iteratively identified the optimal causal structure across participants during our olfactory fMRI paradigm (Gates & Molenaar, 2012). Of note, EC refers to directed interactions between brain regions, while functional connectivity (FC) refers to synchrony among brain regions (Karunanayaka et al., 2014). The modulations of EC due to olfactory ability (i.e., UPSIT scores) were investigated using two‐way analysis of covariance (ANCOVA). Modulations of EC have shown to be related to olfactory‐related behavioral responses (Karunanayaka et al., 2017). This analysis is in line with our goal of exploring network integrity measures to improve olfactory prediction of cognitive ability in AD.

## 3 RESULTS

### 3.1 Demographic and cognitive comparisons

Behavioral tests scores in Table 1 (MMSE, CVLT‐II, DRS‐2, and UPSIT) showed significant group differences (one‐way ANOVA, *p* < 0.0001). The MCI group exhibited a higher variability in neurocognitive and olfactory scores, overlapping with low performing CN and high performing AD subjects. UPSIT scores were positively correlated with cognitive test scores across all subjects (CVLT‐II: *r* = 0.66, *p* < 0.0001; DRS‐2: *r* = 0.73, *p* < 0.0001; MMSE: *r* = 0.70, *p* < 0.0001). UPSIT and cognitive test scores were also correlated when both AD and MCI samples were combined (CVLT‐II: *r* = 0.45, *p* = 0.0054; DRS‐2: *r* = 0.50, *p* = 0.0018; MMSE: *r* = 0.56, *p* = 0.0003), suggesting a strong association between olfactory and cognitive functions.

### 3.2 ON and DMN activity comparison

**Figure 2:**
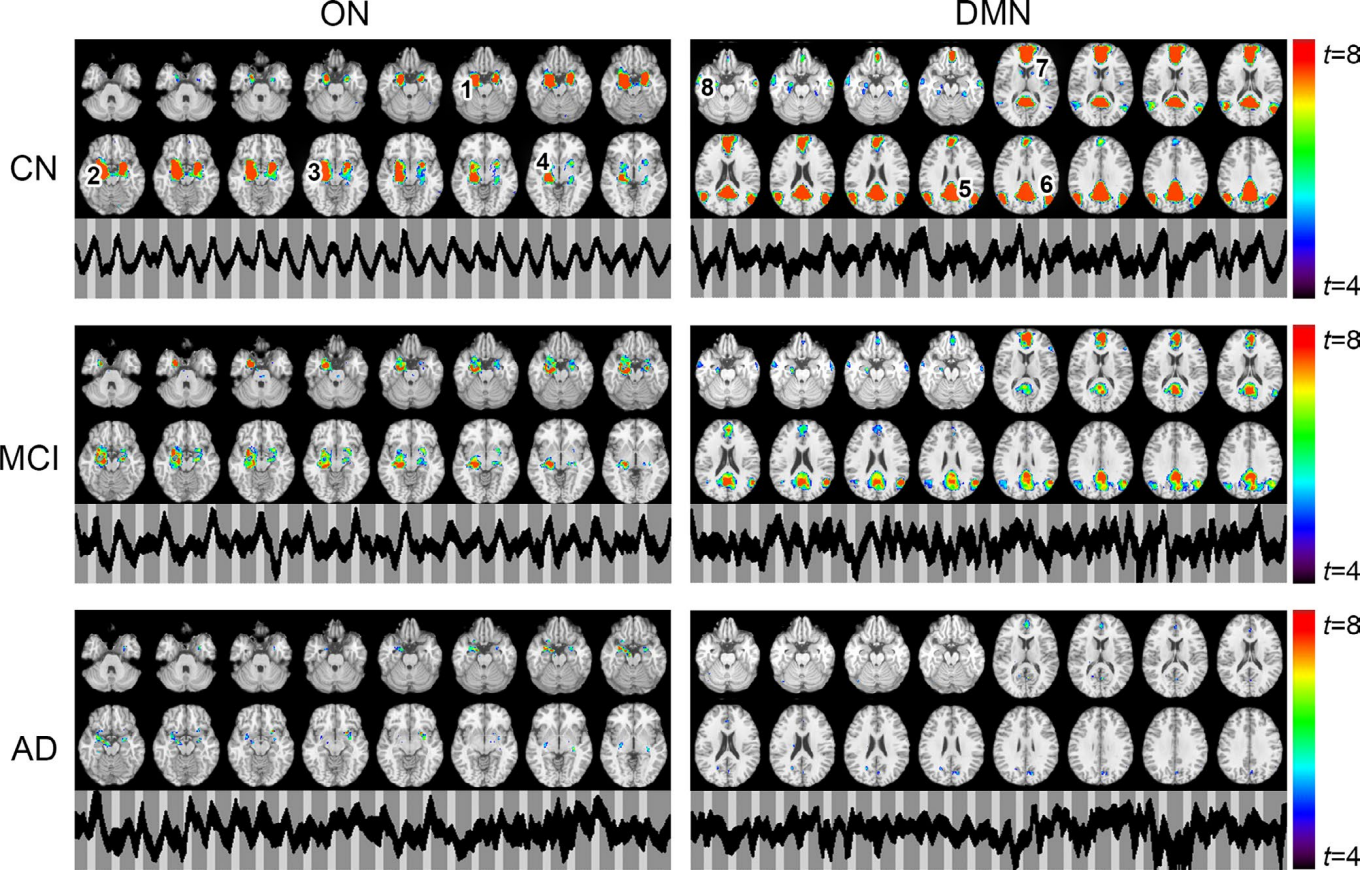
(a) The spatial and temporal behavior of the olfactory network (ON) and the default mode network (DMN) during the olfactory fMRI paradigm in cognitively normal (CN), mild cognitive impairment (MCI), and Alzheimer's disease (AD) subjects. Note that in both networks, the spatial extent of activation progressively diminishes in MCI and AD. Likewise, the task‐related temporal behavior of ON is progressively disrupted in MCI and AD. ON: 1, primary olfactory cortex (POC); 2, hippocampus, 3, insula, and 4, striatum. DMN: 5, posterior cingulate cortex (PCC), extending dorsally into the precuneus; 6, bilateral inferior parietal cortex (IPC, left and right); 7, medial prefrontal cortex (mPFC)/anterior cingulate cortex; and 8, medial/lateral temporal lobe (MTL)

The ON was identified by gICA in AD, MCI, and CN samples as shown in Figure 2, which encompassed the primary olfactory cortex (POC), hippocampus, insula, and striatum (Karunanayaka et al., 2014). Resembling the resting state DMN, gICA identified a modulated DMN from our olfactory fMRI data, which encompassed (a) the posterior cingulate cortex (PCC), extending dorsally into the precuneus; (b) the bilateral inferior parietal cortex (IPC, left and right); (c) the medial prefrontal cortex (mPFC)/anterior cingulate cortex; and (d) the medial/lateral temporal lobe (MTL). When spatial maps for both ON and DMN were compared across groups, both networks showed diminished activation in MCI subjects, while significantly less activation was observed in AD subjects. Figure 2 also includes the corresponding temporal behavior of each network. In CN, ON, and DMN revealed periodic signal changes that followed the olfactory fMRI task timing structure. These fluctuations became lower in magnitude and divergent (spread out) in MCI and AD subjects.

**Figure 3:**
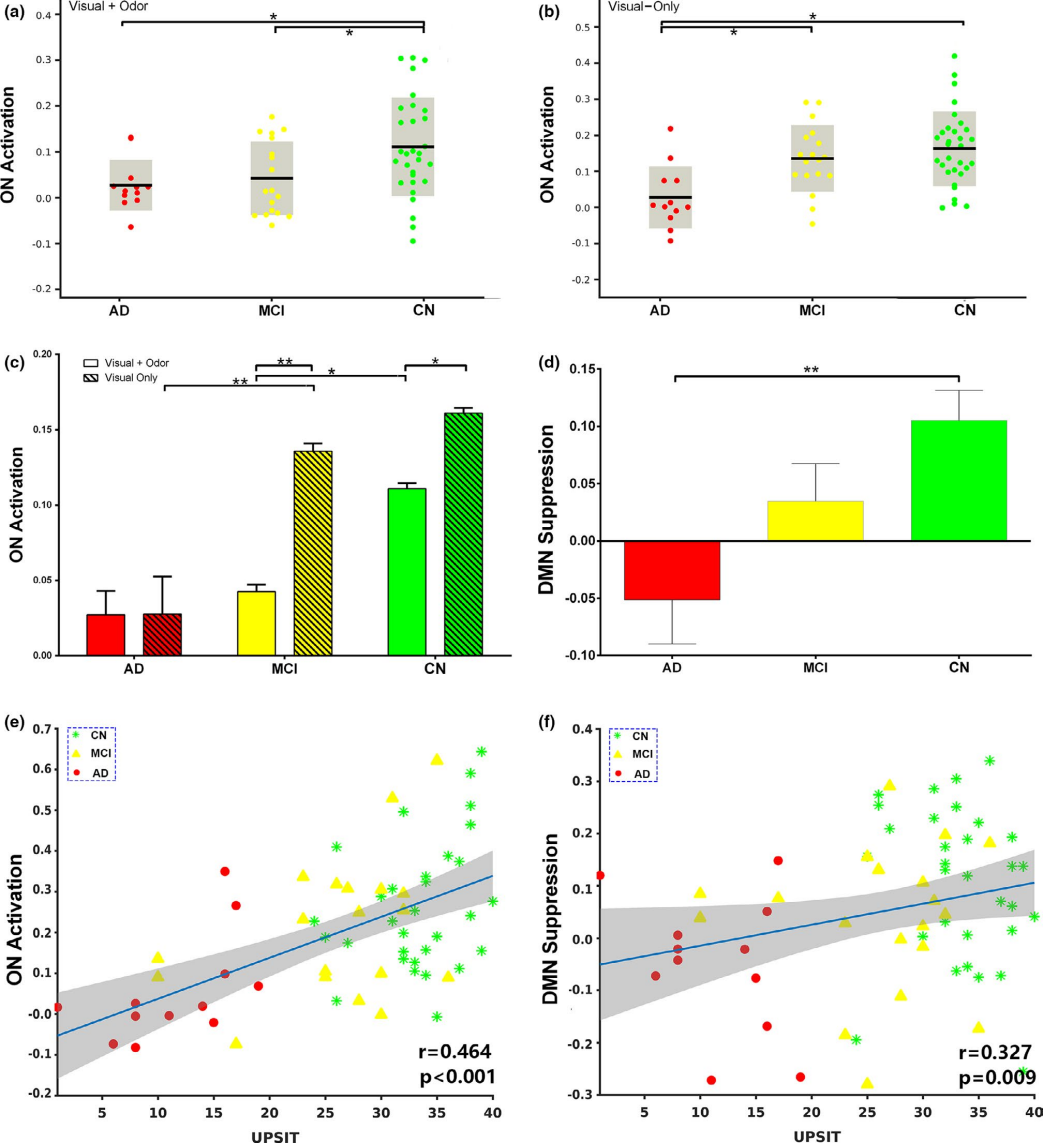
(a) olfactory network (ON) activation during odor+visual conditions in Alzheimer's disease (AD), mild cognitive impairment (MCI) and cognitively normal (CN). Odor+visual condition involves sensory processing of odors. Of note, we removed two outliers using the criterion activation > mean ± 2.5 *SD* from this analysis. Our data driven method of fitting a group HRF (Figure 4) to each subject prohibited reliable estimation of activation in these two subjects. (b) ON activation during visual‐only conditions in AD, MCI and CN. As shown in Karunanayaka et al. (2015) visual‐only conditions are subserved by higher‐order cognitive processes. MCI show a unique pattern of activation with respect to AD during odor+visual and visual‐only conditions (c) ON activation differences between odor+visual and visual‐only conditions in AD, MCI and CN. MCI behavior closely resemble CN. In contrast, no significant differences were found between the two conditions in AD. Additionally, the difference between odor+visual and visual‐only conditions in MCI is greater compared to CN because of low level activation during sensory processing of odors, that is, ordor+visual condition, in MCI. Thus, the patterns of ON activation supports compensatory mechanisms in MCI. *p < 0.05; **p < 0.01. (d) Impaired default mode network (DMN) suppression (or deactivation) in AD. Of note, DMN suppression in AD is not significantly different from MCI. (e) Correlation between the ON activation and University of Pennsylvania Smell Identification Test (UPSIT) scores in AD, MCI and CN. (f) Correlation between DMN suppression and UPSIT scores in AD, MCI and CN. *p < 0.05; **p < 0.01

We also quantified ON activation during odor+visual and visual‐only conditions in terms of average, single‐trial β estimates shown in Figure 3a,b. Significant differences in β estimates for odor+visual condition were observed between MCI and CN (Figure 3a). In contrast, significant differences in β estimates for visual‐only conditions were observed between AD and MCI samples (Figure 3b). Additionally, significant differences in β estimates between odor+visual and visual‐only conditions were observed in MCI and CN (Figure 3c). Lastly, the difference between odor+visual and visual‐only activation was much higher in MCI compared to CN. However, no significant differences were detected between estimates of odor+visual and visual‐only conditions in the AD group. Of note, these group differences persisted after covering MMSE scores to control for subjects’ overall cognitive performance.

**Figure 4:**
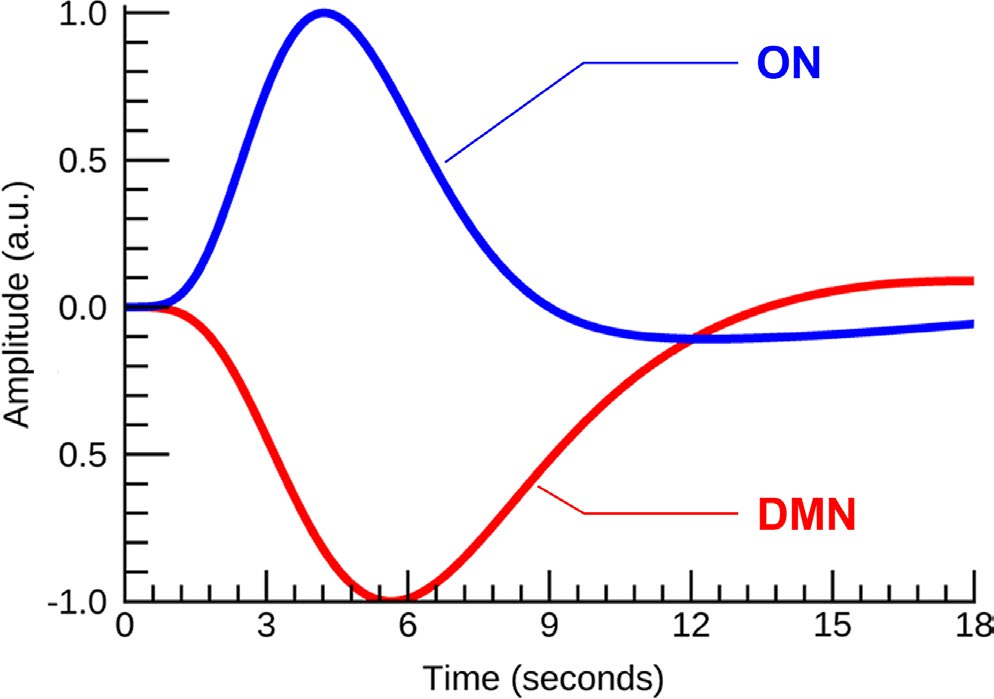
The hemodynamic response functions (HRFs) of default mode network (DMN) and olfactory network (ON) in cognitively normal (CN) during the olfactory fMRI paradigm. They are in opposite phase. This phase reversal becomes progressively disrupted in mild cognitive impairment (MCI) and Alzheimer's disease (AD)

Figure S1 shows the average ON activation during odor+visual and visual‐only conditions. This is a further analysis of Figure 3a,b. Significant differences in single trial β estimates at *p* < 0.01 were found between the AD and MCI, and AD and CN, indicating that ON is compromised but functional in MCI, and dysfunctional in AD. Prior to calculating ON activation during odor+visual and visual‐only conditions, the ON and DMN HRFs were estimated using IC time courses shown in Figure 2. Corroborating previous results from a young healthy cohort in Karunanayaka et al. (2017), HRFs for the CN group showed a 180° phase difference between ON and DMN during our fMRI task, that is, when ON was activated, the DMN was suppressed (Figure 4). Thus, task‐related DMN activation is represented as a negative signal change, termed *suppression*. This CN HRF was used to estimate ON activation and DMN suppression in MCI and AD subjects**.** The random nature of time courses shown in Figure 2 prohibited reliable estimation of HRFs in AD and MCI subjects. Of note, this random behavior of the fMRI signal in AD and MCI is consistent with known abnormal low frequency signal fluctuations and hyper/hypo‐FDG activity in olfactory and parietal regions in AD (Song et al., 2013; Zhou, Yu, Duong, & Initiative, 2015).

Figure 3d reveals the extent of the DMN suppression (or deactivation) during stimulation epochs. Significantly lower DMN suppression was found for AD vs. CN. Neither the level of DMN suppression nor the ON activation shown in Figure S1 was correlated with UPSIT scores in AD, MCI, or CN subjects alone. However, the ON activation and the level of DMN suppression was positively correlated with UPSIT scores in the combined group, likely reflecting the effects of AD pathology (Figure 3e,f).

### 3.3 Olfactory and memory score comparisons

**Figure 5:**
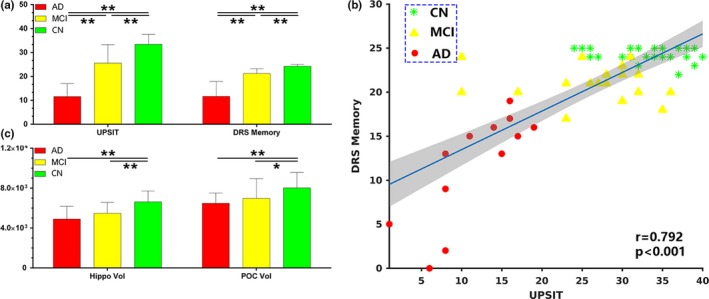
(a) University of Pennsylvania Smell Identification Test (UPSIT) and DRS‐memory scores in Alzheimer's disease (AD), mild cognitive impairment (MCI) and cognitively normal (CN). (b) Correlation between the UPSIT and DRS‐memory scores in the combined group. (C) Volumes of the hippocampus and primary olfactory cortex (POC) in AD, MCI and CN. *p < 0.05; **p < 0.01

Figure 5a shows UPSIT and DRS‐memory score comparisons between AD, MCI, and CN. Both measures showed significant group differences at *p*‐value < 0.01. Only in AD were UPSIT scores correlated with DRS‐memory scores (*r* = 0.79 and *p* < 0.002). However, in the combined group shown in Figure 5b, UPSIT scores were correlated with DRS‐memory scores, likely reflecting the effects of AD.

### 3.4 POC and Hippocampal volume comparisons

Figure 5c shows the comparisons for the hippocampal and POC volumes between groups. Significant brain atrophy was observed in the hippocampus (post hoc *t* test, *p* < 0.01) and POC (post hoc *t* test, *p* < 0.01) in AD and MCI samples. Although AD subjects showed lesser hippocampal and POC volumes compared to MCI, the differences did not reach statistical significance. The POC volumes were significantly correlated with UPSIT scores (*r* = 0.32, *p* < 0.001) and DRS‐memory scores (*r* = 0.5, *p* < 0.001) in the combined group. Similarly, the hippocampal volumes were significantly correlated with UPSIT (*r* = 0.56, *p* < 0.001) and DRS‐memory (*r* = 0.56, *p* < 0.001) scores in the combined group.

### 3.5 Correlation between the EC, UPSIT and DRS‐memory scores in MCI

**Figure 6:**
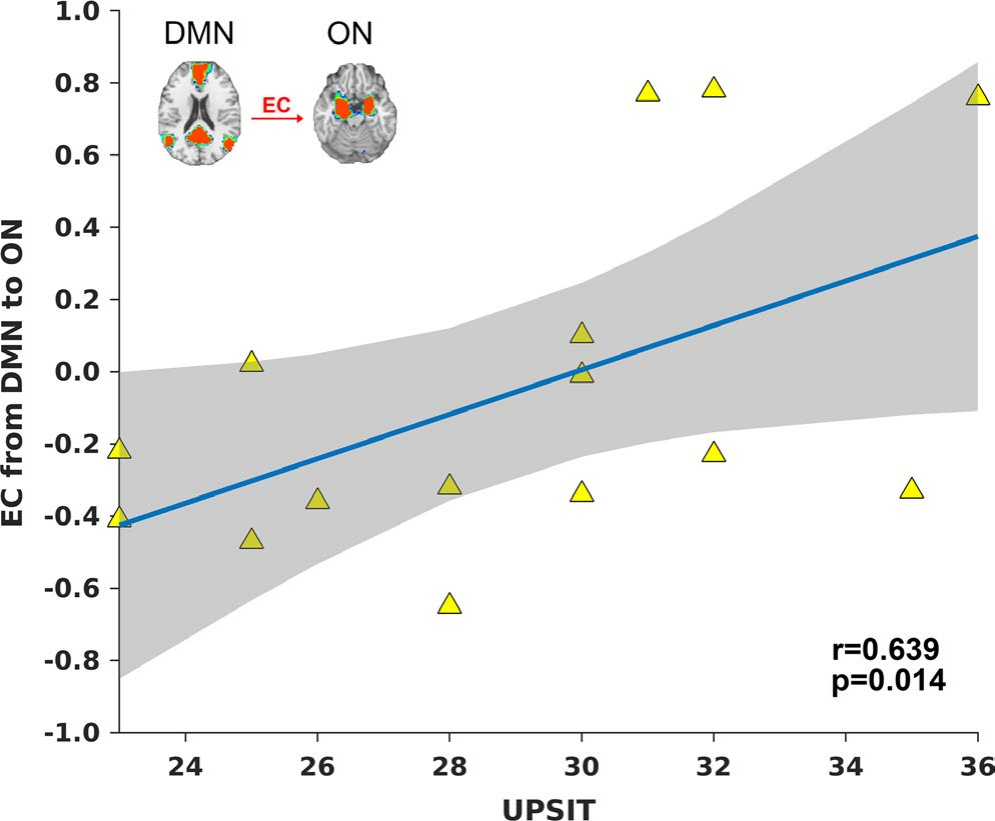
The effective connectivity (EC) between the default mode network (DMN) and olfactory network (ON) augment olfactory performance. The age corrected EC during the fMRI task predicted olfactory performance in mild cognitive impairment (MCI) subjects. A Significant University of Pennsylvania Smell Identification Test (UPSIT) × DRS‐memory interaction effect (*p* < 0.05) on the EC was also observed. Of note, each triangle represents one subject

In order for network integrity to be a useful measure for predicting cognitive ability based on olfactory performance in AD, we investigated the ON‐DMN connectivity in MCI, which is demonstrated to be a highly dynamic group. As such, we expected ON‐DMN connectivity to be highly correlated with UPSIT scores in MCI subjects who are hyposmic and anosmic. Figure 6 shows the EC from DMN to ON in the MCI sample. It is positively correlated with UPSIT scores (*r* = 0.639, *p* = 0.014). Of note, we only included MCI subjects who were hyposmic or normosmic, that is, UPSIT score was >20. This EC was not correlated with the DRS‐memory sores. A two‐way ANCOVA was also performed with UPSIT and DRS‐memory scores as independent covariates. This analysis investigated whether the influence of olfactory function on EC is dependent on memory function. A significant UPSIT × DRS‐memory interaction effect (*p* < 0.05) on the EC (between the DMN and ON) was also identified. Therefore, the EC between the ON and DMN has the potential to serve as a sensitive biological measure linking olfactory and memory deficits in AD.

## 4 DISCUSSION

**Figure 7:**
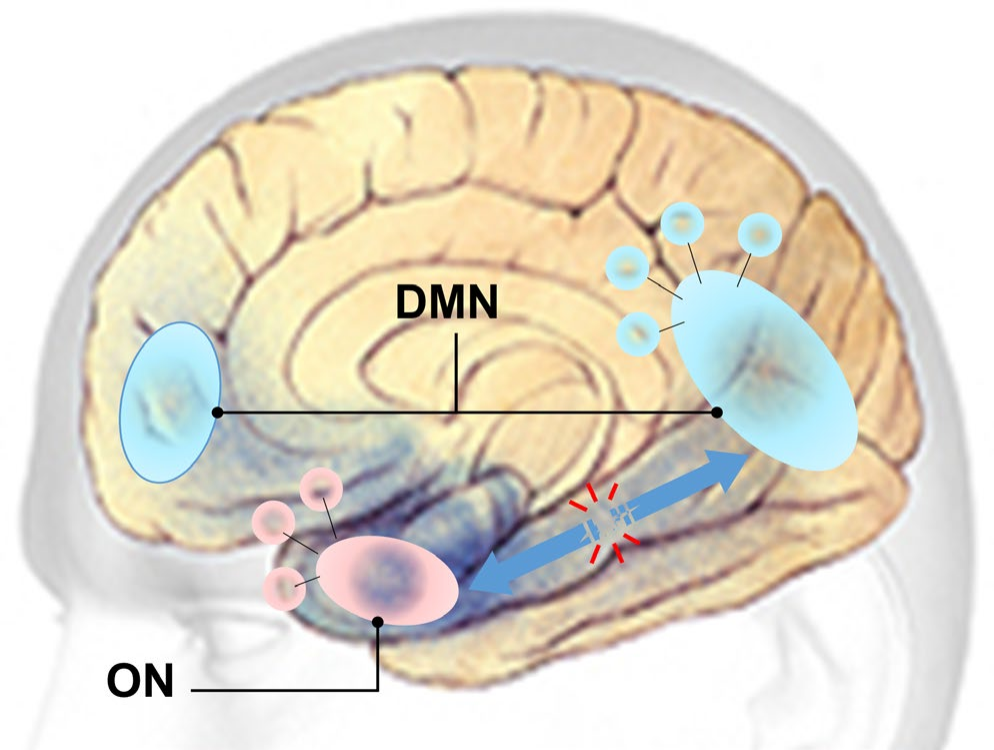
Alzheimer's disease (AD) neurodegeneration model linking olfactory deficits to dementia. AD pathology compromises the integrity of hippocampus causing deficits in effective connectivity (EC) between the olfactory network (ON) and default mode network (DMN). Critically, this model provides a testable hypothesis to relate AD neurodegeneration‐to‐olfactory/memory impairment. It provides pathophysiologic insight into neurodegenerative processes and links olfaction to memory and other cognitive‐deficits

Overall findings of this study elucidated links between olfaction, memory and neurodegeneration in terms of activity and connectivity of the ON and DMN. The DMN suppression during the olfactory fMRI task may signal the allocation of neural resources (via DMN‐ON EC) to meet the cognitive demands of olfactory‐related processing (Anticevic et al., 2012; Karunanayaka et al., 2017). This hypothesis is supported by previous research highlighting the functional relevance of DMN suppression during goal directed and externally oriented task performance (Anticevic et al., 2012). Our results suggest that AD‐related olfactory deficits are most likely caused by disruption to the ON and its connection to the DMN (Vasavada et al., 2017). Evidence from previous fMRI studies supports a functional connection between the ON and DMN via the hippocampus (Raichle, 2015a). Therefore, in Figure 7 we propose a mesoscale brain network model that functionally and anatomically links the ON to the DMN via the hippocampus (Karunanayaka et al., 2017). Neuronal loss in prodromal AD can disrupt the ON‐DMN network, but how this translates into olfactory and other cognitive impairments remains unclear. This model will provide a solid theoretical framework to further our understanding of AD onset and progression. It may also contribute to identifying individuals who are at risk of developing AD and characterizing the effectiveness of interventions, which are crucial steps in developing treatment to be applied before substantial neurological compromise.

Previous research provides clear support for the proposed network's involvement in odor processing. For instance, Martin, Beshel, and Kay (2007) showed that the hippocampus is part of the network that subserves odor‐discrimination learning (Martin et al., 2007). Similarly, Gourévitch, Kay, and Martin (2010) detected a strong unidirectional functional coupling between the olfactory bulb (OB) and hippocampus (Gourévitch et al., 2010). Since OB is connected to POC, the network formed by the POC and hippocampus represents a viable framework to assess functional links between olfaction and higher‐order cognition supported by the hippocampal formation (Gottfried, 2010). It is well known that early AD pathology damages the POC and the hippocampus (Braak & Braak, 1996; Braak, Braak, Bohl, & Bratzke, 1998). Our findings suggest that this early pathology disrupts the integrity of the EC between the ON and DMN. It is likely that this disruption leads to olfactory deficits with concurrent memory deficits. Together, these findings are in line with the notion that neurodegeneration in AD spreads across functional networks (Lehmann et al., 2013). Thus, the proposed model may have the ability to differentiate patterns of olfactory deficits and their trajectory, leading to new studies focusing on coupling impairments as the main cause of brain network dysfunction in AD progression (Filippi et al., 2017). These results suggest that understanding network integrity, combined with olfactory performance (e.g., UPSIT), may provide a more precise measure to improve prediction of cognitive ability in AD**.**

### 4.1 DMN suppression

A substantial body of evidence on DMN provides strong support for the hypothesis that DMN activity underlies internally‐directed thought, and that this activity is suppressed during cognitively demanding tasks requiring externally‐oriented cognition(M. D. Fox et al., 2005; Raichle, 2015b). The level of DMN suppression has also been shown to be proportional to the cognitive load of the task (Anticevic et al., 2012). The observed DMN suppression in this study, therefore, may signal the decoupling of internally directed thought to facilitate the allocation of mental and neural resources to olfactory task processing (Anticevic et al., 2012). Alternatively, differences in the level of DMN suppression could be interpreted as differences in influence or modulation between the DMN and ON (Karunanayaka et al., 2017). Thus, quantifying DMN suppression, a phenomenon characterized by rich cognitive activity, may provide a basis for developing potential biomarkers to characterize AD as well as at‐risk populations such as MCI and asymptomatic APOE‐ε4 allele carriers (Filippini et al., 2009).

The DMN and other resting‐state networks are flexible and dynamic, and their behaviors are modulated during externally oriented task performance (Deco, Jirsa, & McIntosh, 2011). For example, in the CN group the DMN behavior is anti‐correlated with the ON behavior during odor processing (Karunanayaka et al., 2017). Previous resting state studies of AD have provided accumulating evidence for disease‐related alterations within the DMN (Terry, Sabatinelli, Puente, Lazar, & Miller, 2015). In line with this finding, the DMN spatial activity during olfactory processing was found to be at significantly lower levels in MCI and AD subjects compared to CN (Figure 2). Furthermore, the DMN suppression during odor processing demonstrated progressive reduction (more so in AD than MCI) in AD and MCI (Figure 3d). Our results, therefore, can be interpreted as reflecting a difficulty related to switching from a “resting‐state” to a “task‐active state” of brain function in AD. This could be due to a failure of brain regions in the DMN to establish rapid and efficient synchronization in their activity. Since we found an EC from the DMN to the ON in MCI that is correlated with UPSIT, we hypothesize that this connectivity reflects the ON's ability to recruit cognitive resources utilized by the DMN for olfactory processing. This is supported by DMN's known engagement in broad‐based continuous sampling of external and internal environments (Raichle et al., 2001). For example, incoming olfactory stimuli might result in DMN suppression, thus releasing DMN resources to allow ON to switch from a resting‐state to a task‐active state (Karunanayaka et al., 2017; Raichle, 2015a; Raichle et al., 2001).

The positive correlation between EC and UPSIT in hyposmic and normosmic MCI subjects (i.e., those with an UPSIT score greater than 20) underscores the significance of ON‐DMN connectivity for olfactory functioning (Karunanayaka et al., 2017). A significant UPSIT × DRS‐memory interaction effect was also found on the EC between the DMN and ON. Note that MCI subjects exhibited large variations in neurocognitive, olfactory and neural measures, overlapping with CN and AD subject scores. Conversely, the tight range of UPSIT scores in CN subjects likely contributes to the lack of correlation between EC and UPSIT within that group. Suppression of DMN and its EC to ON is important because anatomic as well as functional imaging studies have repeatedly shown tight connections between DMN and the temporal lobe hippocampal regions implicated in memory consolidation (Dennis & Thompson, 2014). Therefore, ON, DMN, and the DMN‐ON EC can potentially provide a mechanistic avenue to assess and quantify early stage AD‐related functional degeneration when the EC is compromised but not yet fully disrupted.

### 4.2 ON activation

The odor–visual association paradigm produced significantly different ON activation patterns between AD and MCI. Specifically, the activation level in MCI during odor+visual conditions, where sensory processing of odorants was involved, had reached the same level of decline as AD subjects (Figure 3c). Thus, the sensory aspect of odor in MCI appears to be disrupted. This is in contrast to significantly different UPSIT scores in respective cohorts (Figure 5a). The odor+visual activation pattern (Figure 3c) in MCI and CN is in agreement with that of POC and Hippocampal tissue volumes in respective groups (Figure 5c). That is, there are significant differences in ON activation between MCI and CN with concurrent differences in the POC and Hippocampal volumes. However, visual‐only activation pattern (Figure 3c) in MCI and CN is not in agreement with that of POC and Hippocampal tissue volumes in respective groups (Figure 5c). That is, there are no significant differences in visual‐only activation between MCI and CN although showing significant differences in the POC and Hippocampal volumes. Furthermore, during visual‐only conditions, significantly different activation levels were observed between MCI and AD, paralleling UPSIT scores in the respective cohorts. Thus, olfactory fMRI seems to be a sensitive marker for early detection as far as AD is concerned (Vasavada et al., 2015). Previously, we have proposed that visual‐only conditions in this fMRI task are subserved by persistent attention, goal‐oriented attention and working memory as part of the odor–visual pairing process (Karunanayaka et al., 2015). An alternative interpretation for visual‐only activation is to consider them as a difference in learned expectation within the predictive coding framework in which the brain is constantly attempting to predict incoming signals, both in terms of predictions and prediction errors (Karunanayaka et al., 2017; Rao & Ballard, 1999). Thus, two important conclusions can be made based on our results: (a) ON activity in MCI is not completely affected by AD pathology. This is because the differential fMRI activation pattern between odor+visual and visual‐only condition is still similar to CN, suggesting that MCI subjects may be using compensatory mechanisms for olfactory processing, that is, the brain's ability to recruit other regions as resources during olfactory task performance (Buchsbaum et al., 1991; Raichle et al., 1994) and (b) from a clinical perspective, the ON activation profile may offer a more sensitive functional imaging marker for the early detection of MCI than volumetric measurements.

It is important to note that our fMRI task is not a simple olfactory detection paradigm. It involves rapid olfactory learning, manipulating odor‐visual associations, and stimulus expectation (Karunanayaka et al., 2015). The relational memory component in this task is different from that of a typical odor‐visual association task. Typically in such a task, an odor is associated with a visual stimulus and that association is probed later. Nevertheless, as described in Karunanayaka et al. (2015), pairing of visual and olfactory stimuli in this task is rapid and enhances fMRI activation in the POC and significantly improves the reliability of the olfactory fMRI. In summary, our fMRI task is a simple and effective one with minimal cognitive confounds and associated variability increasing the feasibility of using fMRI in preclinical and early stage AD studies. Since olfactory deficits occur at an early stage of AD disease progression, before significant memory and cognitive impairments emerge, olfactory testing in AD, may offer a simple and effective tool to assess AD pathology that can be developed into a useful biomarker in clinical settings.

Although olfactory deficits are prevalent in MCI, only a fraction of them (about 10%–15% annually) convert to AD (Petersen et al., 2001). However, olfactory identification deficits are known to be associated with a four to fivefold increased risk of converting from MCI to AD. Olfactory testing, therefore, can be useful to improve diagnostic as well as predictive accuracy of MCI conversion into AD (Devanand et al., 2008). According to the pathophysiological viewpoint presented in this paper, connectivity between brain regions, within and between the ON and DMN, might be aberrant to a greater extent in MCI converters. For instance, there is evidence for impaired FC in AD between the posterior cingulate cortex (PCC) and the hippocampus, probably as a consequence of early structural alterations in the hippocampal formation (Schultz et al., 2017; Vannini et al., 2017). The hippocampus, while not part of the core ON, is nonetheless involved in olfactory processing (Gottfried, 2010). Lack of longitudinal fMRI and cognitive data investigating the utility of FC in predicting olfactory and cognitive performance in AD is a limitation of this study. Future studies must also attempt to delineate aging effects in FC and EC, a critical demographic risk factor for olfactory decline (Masurkar & Devanand, 2014).

### 4.3 Conclusion and future directions

The disruption in both the ON and DMN due to neurodegeneration is one of the most likely causes of AD‐related olfactory deficits. Shifting from evaluating focal pathology to the assessment of network integrity, we conclude that olfactory deficits in AD cannot be fully explained by a primary dysfunction in the backbone of the “ON” proper. This is corroborated by the observation that AD neurodegeneration causes disruption between ON and other multi‐modal networks such as the Salience Network (SN) and Dorsal Attention Network (DAN) (Schultz et al., 2017). Nevertheless, our quantitative network integrity framework is not merely descriptive, it provides pathophysiologic insight into neurodegenerative processes. Furthermore, by direct juxtaposition of functional (i.e., odor+visual and visual‐only conditions) and structural patterns of neurodegeneration, we provided empirical evidence for differential disease mechanisms in MCI patients. These results direct future research toward an integrative approach in studying ON dysfunction in AD. This approach may be critical to chisel out the disconnection pathophysiology potentially leading to AD dementia (Delbeuck, Van der Linden, & Collette, 2003; Lacalle‐Aurioles et al., 2016). In sum, olfactory fMRI and behavioral testing when coupled with the ON‐DMN network model will provide a unique opportunity to directly and noninvasively address the functional consequences of neuropathological changes in the AD brain. Understanding the dynamic behavior of the DMN during olfactory processing may provide a new frontier in olfactory research where the selective anatomic vulnerability in AD is replaced by the syndromic‐specific network vulnerability (Guo et al., 2016). Once discriminatory connectivity signatures are established for the ON in AD, subsequent detection and evaluation of AD can be augmented with a validated olfactory test, driving clinically important network differences that may influence the monitoring and treatment of AD patients (van der Burgh et al., 2017; Welsh, Jelsone‐Swain, & Foerster, 2013).

## CONFLICT OF INTEREST

The authors report no competing interests.

## Data Availability

The data that support the findings of this study are available on request from the corresponding author. The data are not publicly available due to privacy or ethical restrictions.
