# Supplementary figures and images for: Disruptions of the olfactory and default mode networks in Alzheimer's disease
Source: Brain Behav. 2019 Jun 4;9(7):e01296. doi: 10.1002/brb3.1296 (PMC6625464; doi:10.1002/brb3.1296)

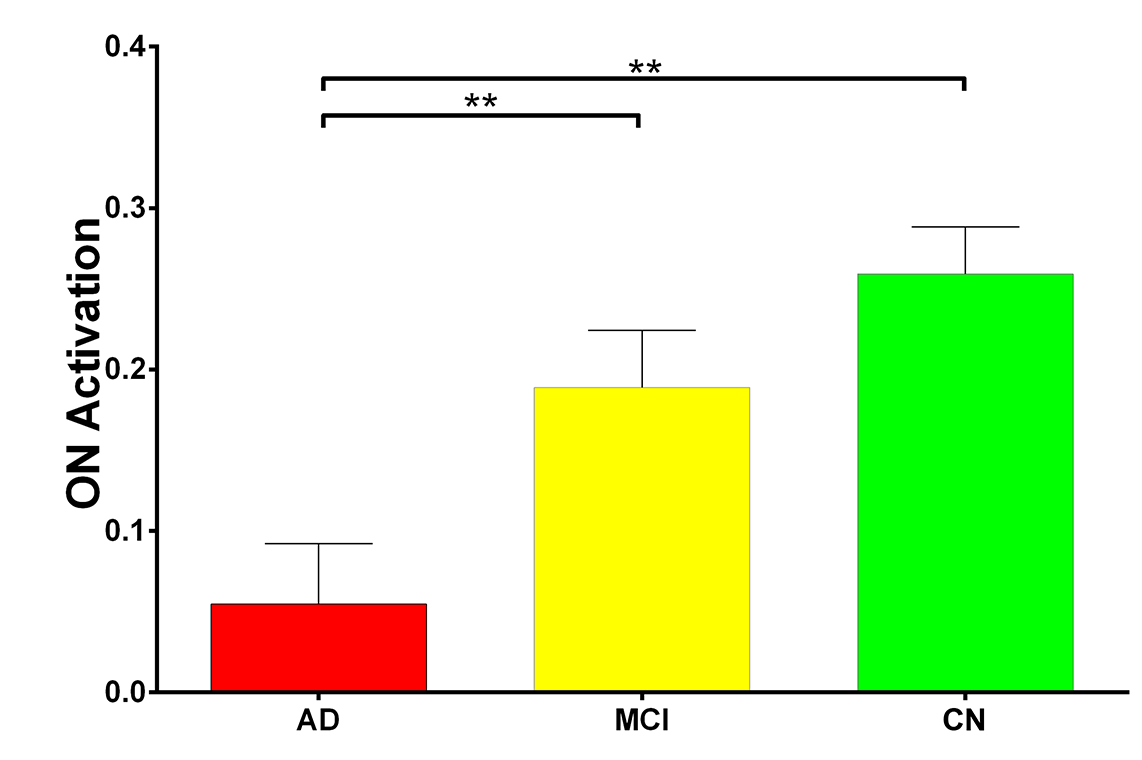

Supplement: Supplementary file 1 [file BRB3-9-e01296-s001.tif]
